# Editorial: *C. elegans* host-microbiome interactions: From medical to ecological and evolutionary model

**DOI:** 10.3389/fcimb.2022.1035545

**Published:** 2022-09-26

**Authors:** Michael A. Herman, Javier E. Irazoqui, Buck S. Samuel, Nic Vega

**Affiliations:** ^1^ School of Biological Sciences, University of Nebraska-Lincoln, Lincoln, NE, United States; ^2^ Department of Microbiology and Physiological Systems, University of Massachusetts Medical School, Worcester, MA, United States; ^3^ Alkek Center for Metagenomics and Microbiome Research and Department of Molecular Virology and Microbiology, Baylor College of Medicine, Houston, TX, United States; ^4^ Department of Biology, Emory University, Atlanta, GA, United States

**Keywords:** host-microbe interactions, microbiome, *C. elegans*, host-pathogen interactions, microbiome & dysbiosis

## Introduction and editorial

Microbiomes often form specific functional associations with their hosts. Correlations between microbiome membership and states of host health and disease abound in many systems. However, there are few systems that allow for in depth functional studies that include precise manipulation and interrogation of both microbiome composition and host function. Recently the nematode *Caenorhabditis elegans* - an excellent genetic model organism for studying many fields of biology, including neurobiology and behavior, development, cell biology, and innate immunity - has proven to be a robust system to probe microbiome interactions and their effect on host physiology.

Pioneering research on *C. elegans*-pathogen interactions began over 20 years ago and has led to an in-depth understanding of innate immune pathways, many of which are conserved in other animals. More recent efforts have elucidated that *C. elegans* acquires a diverse and distinctive intestinal bacterial community from its natural habitats of rotting organic matter, and that this community affects the life history, development, behavior, and healthspan of the host (reviewed in Radeke and Herman, 2021).

This Research Topic builds on recent work that has begun to characterize the native microbiome and has identified a common set of bacteria found in the microbiome of *C. elegans*. While some of these bacteria have been shown to be beneficial to the health of *C. elegans*, others can be detrimental, leading to a complex, multi-faceted understanding of host-microbial interactions. *elegans*. Several themes emerge from the papers contributed to this special research topic. These include:

Innate and learned behavioral preferences for beneficial microbes, as a common and lineage-specific feature of C. elegans-microbe interactions
Commensal-mediated alteration of life history and behavior, in mono-association and in the company of pathogens
Microbe-mediated protection from pathogens (MMP) and its specificity in combinations of host and microbe, as well as variation between sexes and among individuals within a population of hosts
Evolutionary adaptation to pathogens and the signatures thereof, as seen experimentally with and without co-evolution of host and parasite, and as detected from standing genetic variation in immunity-related genes in natural populations of worms

As an introduction to the Research Topic, we highlight how these themes are woven through this collection of papers.

Previous work indicated that certain bacteria isolated from wild and microcosm worm microbiomes can be beneficial, showing positive effects on host development (Samuel et al., 2016; Dirksen et al., 2020; Zhang et al., 2021) and under some conditions protecting the host from subsequent invasion by pathogens (Berg et al., 2016). Pérez-Carrascal et al. find that *C. elegans* can show behavioral preference toward beneficial members of the genus *Pantoea* isolated from worms in soil microcosms; greater colonization ability and host attraction combined in this case to produce preferential gut colonization by a beneficial microbe. Benefits in development rate and pathogen protection were not conferred by an environmental isolate of *Pantoea*, suggesting that positive assortment had occurred between worms and their beneficial bacteria in soil microcosms.

More broadly, behavioral preference and host benefit are frequently lineage-specific. A manuscript by Peterson et al. investigated the bacterial preferences of domesticated and “‘wild” strains toward components of the natural *C. elegans* microbiota. Interestingly, they find that the natural *C. elegans* isolate (MY2079), but not the domesticated isolate (N2), changes its preference toward microbiota isolate *Ochrobactrum vermis* (MYb71) following preconditioning on that specific bacterium. Another by Kissoyan et al. showed that components of the natural microbiota can interact by modulating the effects of pathogens and toxins. They previously showed that two natural isolates, *Pseudomonas lurida* (MYb11) and *P. fluorescens* (MYb115) protect the worm against pathogens *Bacillus thuringiensis* (Bt) and *P. aeruginosa* (Kissoyan et al., 2019). In the current work, they show that these strains are not protective in all contexts. Specifically, they demonstrate that while both *Pseudomonas* strains colonize the gut and provide protection to infection, *P. lurida* increases susceptibility to *Bt* toxin, while *P. fluorescens* had no effect. Thus, it appears that microbiota interactions with each other and the host may differ in a context-dependent manner, adding to the complexity of these important biological associations.

Commensal-mediated protection from pathogens is also part of the evolutionary story. As discussed above, microbe-mediated protection (MMP) from pathogens is well documented in *C. elegans* as in other systems. Further, it is well known that infection by pathogens alters host life history, and that the effects of infection, as well as baseline investment in immunity, differ with sex. In a manuscript by Kloock et al., the authors find that the benefit of a protective microbe (*Enterococcus faecalis*) likewise differs with sex, with feminized hermaphrodite (female) worms benefiting more strongly than males from MMP. Further, males and females responded differently to MMP, with females showing increased survival and increased investment in offspring production, while males increased investment in mate search behaviors. Understanding these sex-specific differences in the effects of beneficial microbes on the host may be important for understanding selection on these interactions, particularly in a polymicrobial context.

The natural microbial milieu of *C. elegans* includes not only bacteria, but also viruses and oomycetes. The *Orsay* virus (OrV) is major viral microbiota that has been studied. Previous work has demonstrated the existence of an Intracellular Pathogen Response (IPR) that functions to mitigate proteotoxic stress from infection by OrV and other intracellular pathogens (Bakowski et al., 2014; Reddy et al., 2017; Osman et al., 2018; Reddy et al., 2019). Here, van Sluijs et al. investigate the effects natural genetic variation within the *pals* genes that function to regulate the IPR response to OrV. They find little genetic diversity worldwide within *pals* gene clusters and what exists is only in a few highly divergent haplotypes. This leads the authors to suggest these genes may be under balancing selection under pressure of viral and other intracellular pathogens within genetically distinct wild *C. elegans* strains. Thus, understanding the role of natural genetic variation in the genes that control *C. elegans* responses to pathogens will continue to be an important avenue of research in this new area.

Guns, germs, and needles are the repertoire deployed by the novel *C. elegans* pathogenic oomycete called *Haptoglossa zoospora* in this interesting study by Grover et al.. The authors isolated pathogenic Haptoglossa, which quickly killed various nematode species, their corpses displaying characteristic thalli. Electron and light microscopy showed tell-tale “gun cells”, which deploy a harpoon to infect nematodes leaving behind no scar. RNA-seq and reporter studies showed similarities between the transcriptional response to *H. zoospora* and those to oomycete *Myzocytiopsis humicola* and microsporidium *Nematocida parisii*, suggesting that they elicit the intracellular pathogen response. Any resemblance to the Alien movies is purely coincidental.

As demonstrated in this Research Topic, guidance from well supported ecological and evolutionary theory and modeling approaches, has allowed the worm to become a powerful experimental system for studying host-associated microbial communities. Lessons learned from this model will inform our understanding of the effect the microbiome has on organismal health more broadly, including humans.

## Author contributions

This Research Topic on *C. elegans Host-Microbiome Interactions: From Medical to Ecological and Evolutionary Model* was initially proposed and set up by MH. All the editors worked collaboratively to decide which papers were accepted or rejected, and each manuscript was subject to review by the panel of editors as well as peer reviewers. This editorial introduction was led by MH. Each member of the editorial team contributed their thoughts and revisions to help craft the published document.

## Acknowledgments

The authors wish to acknowledge the contributions of the *C. elegans* researchers whose work is included in this Research Topic as well as the members of each of their laboratories.

## Conflict of interest

The authors declare that the work was conducted in the absence of any commercial or financial relationships that could be construed as a potential conflict of interest.

## Publisher’s note

All claims expressed in this article are solely those of the authors and do not necessarily represent those of their affiliated organizations, or those of the publisher, the editors and the reviewers. Any product that may be evaluated in this article, or claim that may be made by its manufacturer, is not guaranteed or endorsed by the publisher.
